# Evaluating a Targeted Cancer Therapy Approach Mediated by RNA *trans*-Splicing In Vitro and in a Xenograft Model for Epidermolysis Bullosa-Associated Skin Cancer

**DOI:** 10.3390/ijms23010575

**Published:** 2022-01-05

**Authors:** Katharina Woess, Yuchen Sun, Hanae Morio, Anna Stierschneider, Anna Kaufmann, Stefan Hainzl, Lisa Trattner, Thomas Kocher, Birgit Tockner, Victoria Leb-Reichl, Markus Steiner, Gabriele Brachtl, Andrew P. South, Johann W. Bauer, Julia Reichelt, Tomomi Furihata, Verena Wally, Ulrich Koller, Josefina Piñón Hofbauer, Christina Guttmann-Gruber

**Affiliations:** 1 EB House Austria, Research Program for Molecular Therapy of Genodermatoses, Department of Dermatology and Allergology, University Hospital of the Paracelsus Medical University, 5020 Salzburg, Austria; katharina.woess@vetmeduni.ac.at (K.W.); anna.stierschneider@fh-krems.ac.at (A.S.); an.kaufmann@salk.at (A.K.); s.hainzl@salk.at (S.H.); l.trattner@crcs.at (L.T.); t.kocher@salk.at (T.K.); b.tockner@salk.at (B.T.); v.reichl@salk.at (V.L.-R.); joh.bauer@salk.at (J.W.B.); JReichelt@hamad.qa (J.R.); v.wally@salk.at (V.W.); u.koller@salk.at (U.K.); j.d.pinon@salk.at (J.P.H.); 2 Division of Medicinal Safety Science, National Institute of Health Sciences, Kanagawa 210-9501, Japan; yuchen.s@nihs.go.jp; 3 Laboratory of Clinical Pharmacy and Experimental Therapeutics, School of Pharmacy, Tokyo University of Pharmacy and Life Sciences, Hachioji, Tokyo 192-0355, Japan; morihana@toyaku.ac.jp (H.M.); tomomif@toyaku.ac.jp (T.F.); 4 Department of Internal Medicine III with Haematology, Medical Oncology, Haemostaseology Infectiology and Rheumatology, Oncologic Center, Salzburg Cancer Research Institute-Laboratory for Immunological and Molecular Cancer Research (LIMCR), Cancer Cluster Salzburg, Paracelsus Medical University, 5020 Salzburg, Austria; m.steiner@salk.at; 5 Cell Therapy Institute, Spinal Cord Injury and Tissue Regeneration Center Salzburg (SCI-TReCS), Paracelsus Medical University, 5020 Salzburg, Austria; gabriele.brachtl@pmu.ac.at; 6 Dermatology and Cutaneous Biology, Thomas Jefferson University, Philadelphia, PA 19107, USA; andrew.south@jefferson.edu

**Keywords:** epidermolysis bullosa, squamous cell carcinoma, cancer gene therapy, spliceosome mediated RNA *trans*-splicing, Ct-SLCO1B3, herpes simplex virus thymidine kinase, ganciclovir

## Abstract

Conventional anti-cancer therapies based on chemo- and/or radiotherapy represent highly effective means to kill cancer cells but lack tumor specificity and, therefore, result in a wide range of iatrogenic effects. A promising approach to overcome this obstacle is spliceosome-mediated RNA *trans*-splicing (SMaRT), which can be leveraged to target tumor cells while leaving normal cells unharmed. Notably, a previously established RNA *trans*-splicing molecule (RTM44) showed efficacy and specificity in exchanging the coding sequence of a cancer target gene (Ct-SLCO1B3) with the suicide gene HSV1-thymidine kinase in a colorectal cancer model, thereby rendering tumor cells sensitive to the prodrug ganciclovir (GCV). In the present work, we expand the application of this approach, using the same RTM44 in aggressive skin cancer arising in the rare genetic skin disease recessive dystrophic epidermolysis bullosa (RDEB). Stable expression of RTM44, but not a splicing-deficient control (NC), in RDEB-SCC cells resulted in expression of the expected fusion product at the mRNA and protein level. Importantly, systemic GCV treatment of mice bearing RTM44-expressing cancer cells resulted in a significant reduction in tumor volume and weight compared with controls. Thus, our results demonstrate the applicability of RTM44-mediated targeting of the cancer gene Ct-SLCO1B3 in a different malignancy.

## 1. Introduction

**Figure 1:**
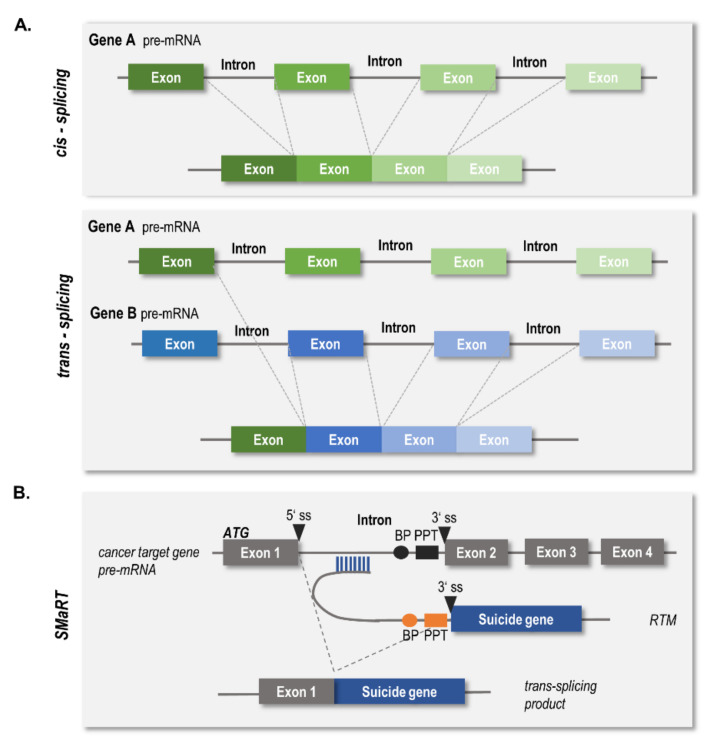
Schematic representation of *cis*- versus *trans*- splicing reactions and SMaRT approach. (**A**) Different types of RNA-splicing. Cis-splicing occurs within one pre-mRNA transcript whereas *trans*-splicing fuses exons from different pre-mRNA transcripts together. (**B**) SMaRT technology in suicide gene therapy approach. An RNA *trans*-splicing molecule targets the intronic region of a cancer target gene thereby inducing a *trans*-splicing reaction and generating a new chimeric RNA transcript consisting of exon1 of the target gene and a suicide gene provided by the RTM. Abbreviations: BD, binding domain; BP, branch point; PPT, polypyrimidine tract; ss, splice site; RTM, RNA *trans*-splicing molecule.

Splicing is a naturally occurring process that takes place during the generation of fully active messenger RNA (mRNA) and is mediated by the cell’s own spliceosome. Herein, noncoding regions (introns) are excised from the pre-mRNA and the coding regions (exons) are fused together to form the mature mRNA. The pre-dominant form in eukaryotic cells is *cis*-splicing, where the exons from one pre-mRNA transcript are joined together (Figure 1A). In contrast, RNA *trans*-splicing involves the joining of exons originating from more than one pre-mRNA transcript. *Trans*-spliced RNA can encode new proteins or non-coding regulatory transcripts, not only resulting in increased proteome complexity, but also contributing to the regulation of gene expression [1].

Although less frequent in humans, RNA *trans*-splicing events have been observed in several physiological and pathological conditions including cancer [2], highlighting a potential role in human health and disease. In prostate cancer e.g., chimeric RNAs have been detected at higher frequency in malignant samples compared to matched healthy tissue [3]. Although detection of these new chimeric transcripts is challenging, they are suggested to have great potential as biomarkers or even as new therapeutic targets [4].

The process of fusing two different mRNA transcripts together by a single *trans*-splicing reaction has also opened up new avenues for RNA reprogramming. In 1999, Putaraju et al. described for the first time a spliceosome-mediated mRNA *trans*-splicing approach (abbreviated “SMaRT”) capable of replacing the 3′ region of the pre-mRNA for the β-subunit of human chorionic gonadotropin gene 6 in a human lung cancer model system [5]. Since then, this technology has been developed further in order to replace 5′- [6,7], 3′- [8,9,10], or even internal exons [11,12] of a target pre-mRNA. While SMaRT technology has been primarily developed for gene therapeutic approaches to replace exons carrying disease-causing mutations (as reviewed by [13]), it has also drawn significant attention as an emerging tool for targeted anti-cancer therapy. The goal of targeted cancer therapies is the elimination of tumor cells while leaving normal cells unharmed. In the case of SMaRT, this has been achieved by *trans*-splicing a suicide gene to a cancer-specific target, thereby enforcing its cell-restricted expression. This approach has successfully been used to deliver the suicide gene thymidine kinase from herpes simplex virus 1 (HSVtk) into cancer cells, rendering cells sensitive to treatment with the prodrug ganciclovir (GCV) [14,15,16,17]. In detail, the HSVtk enzyme catalyzes the phosphorylation of GCV into an active cytotoxin that causes cell death by terminating DNA replication [18].

The *trans*-splicing reaction is facilitated by an artificially engineered pre-mRNA *trans*-splicing molecule (RTM) (Figure 1B) consisting of a binding domain (BD) complementary to intronic regions of a cancer-specific target molecule, splicing elements (BP, branch point; PPT, polypyrimidine tract; 3′ ss, splice site), and the coding sequence of the suicide gene lacking its intrinsic translation start codon. Upon correct *trans*-splicing, a new chimeric transcript is generated, merging the 5′ region of the target mRNA and the suicide gene together. As the translation of the chimeric protein is initiated from the start codon provided by the target mRNA, the specificity of the approach is highly dependent on the cancer-specific expression of the target mRNA [19]. In this respect, cancer-type solute carrier organic anion transporting polypeptide 1B3 (Ct-SLCO1B3) has been shown to be a highly cancer-specific transcript in several different cancers [20,21,22]. Ct-SLCO1B3 is a variant of the liver-type SLCO1B3 (Lt-SLCO1B3) that arises from transcription initiation at an alternate exon 1 (Ct-exon1) located within the large intron 3 of Lt-SLCO1B3. Our previous efforts concentrated on generating a potent RTM (RTM44) able to target Ct-SLCO1B3 and introduce the suicide gene HSVtk into colorectal cancer cells, rendering them sensitive to treatment with GCV [23].

In the present work, we evaluated the efficacy of this specifically designed RTM44 in a different cancer type, cutaneous squamous cell carcinomas (cSCCs), which arise in patients with the rare genetic skin disease recessive dystrophic epidermolysis bullosa (RDEB) [24,25]. RDEB is caused by mutations in the gene *COL7A1*, encoding type VII collagen, the main component of anchoring fibrils that are essential for adhesion of the epidermis to the dermis. As a consequence, patients suffer from blister formation on the skin and mucous membranes, as well as additional comorbidities such as mitten deformities, pseudosyndactyly, and failure to thrive. The overall incidence and prevalence of RDEB is 3.05 and 1.35 per one million live births, as estimated by the U.S. National EB registry [26]. Repetitive cycles of wounding, inflammation, and infection predispose patients to the development of aggressive cSCC, which represents a life-threatening condition in this patient cohort. Epidemiology data from the Australasian EB registry cohort reported a cumulative risk of cSCC development of 76.1% by the age of 35 and a median time to death after first cSCC of 4 years [27].

Importantly, our group has previously shown that Ct-SLCO1B3 constitutes a cancer-specific target gene in RDEB-SCC [23]. By using primers that discriminate between liver- and cancer-type variants, we were able to demonstrate that Ct-SLCO1B3 transcripts were significantly expressed in RDEB-SCC cell lines and patient tumor biopsies as compared with non-tumor RDEB or normal human keratinocyte cell lines, making this variant transcript a potential therapeutic target as well in this tumor entity. Using the same RTM44 as described by Sun et al. [17], we demonstrate here the successful introduction of HSVtk into the coding sequence of Ct-SLCO1B3 in the presence of functional RTM44, and tumor cell killing in cell culture and as well as in a xenograft mouse model for RDEB-SCC following treatment with the prodrug GCV. This study highlights the translatability of a specially designed RTM molecule to different malignancies.

## 2. Results

### 2.1. RTM44 Facilitates the Trans-Splicing Reaction in RDEB-SCC Cells

**Figure 2:**
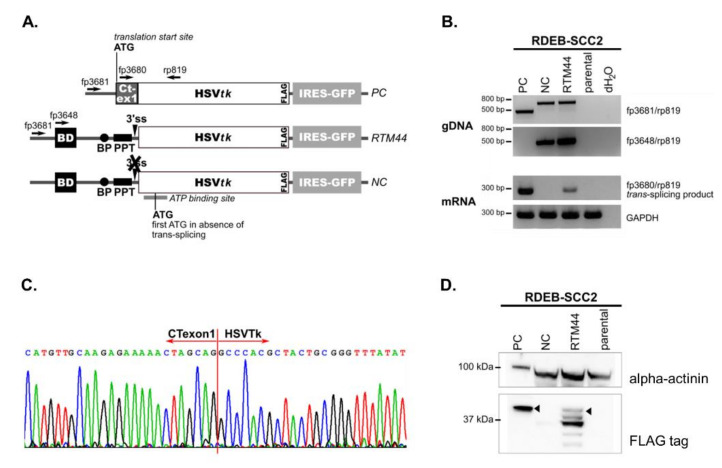
*Trans*-splicing in RDEB-SCC cells. (**A**) RDEB-SCC2 cells were retrovirally transduced with vectors encoding either RTM44, a positive control (PC) expressing the expected fusion transcript or a splicing deficient RTM designated as negative control (NC). (**B**) Correct integration of the various vectors into the host genomic DNA (gDNA) was confirmed by PCR (top panels) using vector- and BD-specific forward primers (fp3681 and fp3648, respectively), as well as an HSVtk-specific reverse primer (rp819). Additionally, we confirmed *trans*-splicing on the mRNA level by sqRT-PCR using primers specific for the expected fusion product (fp3680 and rp819). GAPDH served as control (lower panels). (**C**) Sequencing of the PCR-amplified *trans*-splicing product confirmed accurate *trans*-splicing between the target pre-mRNA and RMT44, resulting in a fusion transcript consisting of Ct-exon1 and HSVtk. (**D**) Detection of *trans*-splicing product on the protein level using an antibody against the FLAG-tag fused to the HSVtk sequence on the C-terminus. Alpha-actinin was used as a loading control. The triangles indicate the fusion protein of Ct-SLCO1B3 and HSVtk (predicted MW of 42 kDa).

In the present study, we utilized RTM44, previously proven to target Ct-SLCO1B3 in a colorectal cancer model. The design and generation of RTM44 has been described in detail by Sun et al. [17]. Briefly, RTM44 consists of a 225-bp BD complementary to intronic sequences immediately following exon 1 of the Ct-SLCO1B3 transcript. RTM44 was further modified to remove any start codons within the BD, as well as cryptic splice sites within a minimal functional HSVtk sequence. This eliminated potential expression of functional HSVtk enzyme directly from the RTM, and reduced undesired splicing within the HSVtk coding region, which could result in a non-functional enzyme. A 3× FLAG-tag incorporated into the C-terminus of the HSVtk sequence facilitated the identification of the chimeric *trans*-splicing product (Figure 2A). In addition to RTM44, we used a positive control vector encoding the expected Ct-HSVtk fusion product (PC), and a negative control vector encoding a splicing-deficient RTM44 (NC) (Figure 2A). All three vectors were used for retroviral transduction of the cancer cell line RDEB-SCC2 established from an RDEB patient [28]. 

We confirmed the integration of the full-length RTM cassette and its variants into the genome of the RDEB-SCC cells by PCR amplification using vector- and BD-specific forward primers (fp3681 and fp3648, respectively) and an HSVtk-specific reverse primer (rp819) (Figure 2B). Using primers specific for the expected fusion product (fp3680/rp819), we performed sqRT-PCR to detect the *trans*-splicing product in total RNA isolated from each cell line. We detected a PCR product of the expected size (206 bp) only in RDEB-SCC lines transduced with the RTM44- and PC- vectors, but not in the parental line or those transduced with the splicing-deficient NC-vector (Figure 2B). Sequencing of the amplified products showed the correct sequence at the expected junction between Ct-exon1 and HSVtk (Figure 2C), thus confirming that accurate *trans*-splicing had occurred.

Expression of the expected fusion protein was confirmed by Western blot analyses of total cell lysates using an anti-FLAG antibody in RDEB-SCC2 (Figure 2D) and in a second RDEB-SCC cell line (Figure S1A). We detected robust expression of an approximately 42 kDa protein, representing the expected fusion product, in cells transduced with the PC vector. Importantly, we detected a similar protein band in RDEB-SCC cells stably expressing RTM44 that was absent in parental cells or those transduced with the NC vector, signifying that this protein was expressed from the *trans*-spliced product. 

We also detected FLAG-tagged proteins of lower molecular weight (MW) in our Western blot analyses, which we further investigated in order to determine their origin (Figure S2). We identified several internal start codons within the encoded HSVtk sequence that could potentially lead to translation of truncated versions of the enzyme directly from the RTM in the absence of *trans*-splicing. Specifically, translation initiation from the first three internal ATGs would result in proteins with predicted MWs of 37.3, 34.5, and 30.7 kDa, respectively, which correspond closely to the observed MWs of the truncated variants detected by Western blot analysis. In order to verify the origin of each HSVtk version, we mutated each of these three start codons in the RTM44 vector separately (MTK1, MTK2, and MTK3), and transfected each into HEK293 cells. These cells lack the expression of Ct-SLCOB3, thereby enabling the evaluation of HSVtk expression and activity in the absence of *trans*-splicing. Western blot analysis of cell lysates confirmed that the 37.3 and 34.5 kDa protein bands were derived from translation initiation at internal ATGs. However, the identity of the proteins with apparent MW of ~30 and ~40 kDa remained undetermined by this analysis. Nevertheless, based on the existing literature, all of these truncated HSVtk variants are expected to lack an intact ATP-binding site required for activity, and thus represent inactive enzymes [29]. Indeed, despite the expression of these truncated HSVtk-related variants in HEK293 cells, the viability of these cells in GCV-killing assays confirmed the lack of activity of any of these proteins.

Taken together, the data confirmed that successful generation of a new chimeric mRNA and active HSVtk-fusion enzyme only occurred when both the functional RTM44 and its Ct-SLCO1B3 pre-mRNA target were present in cells. 

### 2.2. RTM44 Renders RDEB-SCC Sensitive to Ganciclovir Treatment In Vitro

**Figure 3:**
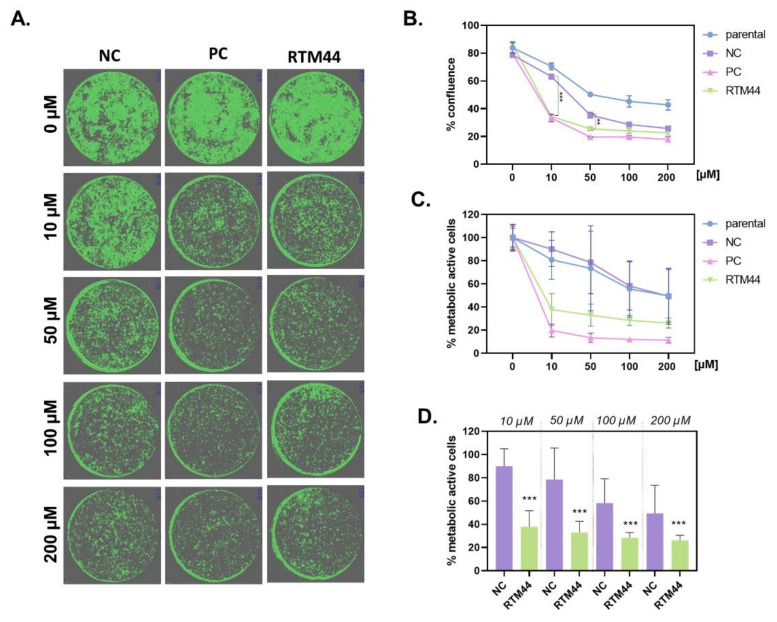
Impact of *trans*-splicing cancer gene therapy on cell viability and confluence in vitro. RDEB-SCC2 cells were stably transduced with the various RTM44 vectors and treated with increasing concentrations of GCV (0—200 µM) for 72 h. (**A**,**B**) Cells were seeded in 96-well plates and cell confluence was measured using life cell imaging (Tecan Spark Cyto). Each experiment was carried out in quadruplicates and the mean ± SD of three experiments are shown. (**C**,**D**) Cell viability was assessed by MTT assay. RTM44 rendered cells sensitive to the treatment of GCV at all concentrations tested compared with the splicing-deficient negative control (NC) or parental cells. Each experiment was carried out in quadruplicates and mean ± SD of four experiments are shown. Statistical analysis: Mann–Whitney test *** *p* < 0.0001.

To demonstrate that RTM44 is capable of rendering tumor cells susceptible to treatment with GCV, parental RDEB-SCC cells and cells stably expressing RTM44, PC, or NC, were treated with increasing concentrations of GCV (10–200 μM) for up to 72 h. Cell confluence and cell viability were then assessed by cell imaging and MTT assay, respectively (Figure 3). We observed a dose-dependent reduction in confluence of cells harboring RTM44 and PC upon GCV treatment (Figure 3A,B). A concentration of 10 µM already showed significant decrease in cell confluence compared with parental and NC-expressing cells. Notably, increasing concentrations of GCV (>50 µM) induced a general cytotoxicity in cells, as demonstrated by significant reduction of cell confluence in NC and parental cells.

These data were corroborated by cell viability measurements, which were significantly decreased in cells harboring RTM44 and PC upon GCV treatment of concentration ≤10 µM when compared with untreated controls (Figure 3C). GCV treatment with 10 μM of RDEB-SCC2 cells transduced with PC and RTM44 constructs displayed a viability of 19.72% and 37.89%, respectively, after 72 h of treatment. In contrast, parental RDEB-SCC2 were largely unaffected (viability of 80.85%), and NC-transduced cells exhibited a viability of 90.02%. At higher concentrations of GCV, reduced metabolic activity in the parental and NC cells was also observed compared with the untreated controls. Similar effects were observed in a second RDEB-SCC cell line (Figure S1B).

### 2.3. RTM44 Shows Efficacy in a Xenograft Mouse Model for RDEB-SCC

**Figure 4:**
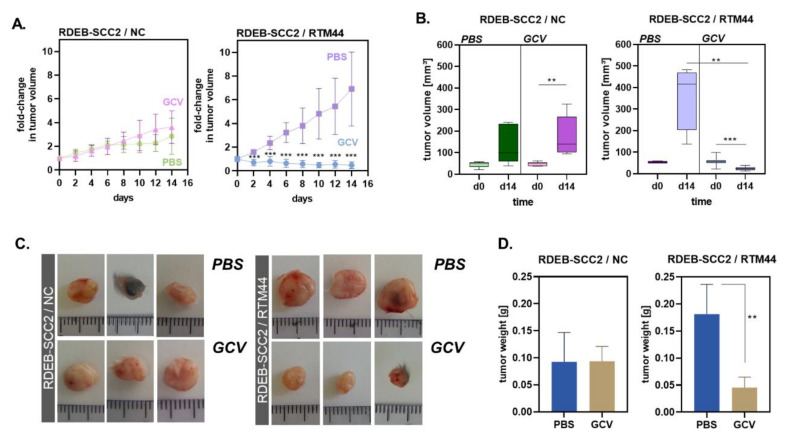
Tumors expressing RTM44 are sensitive to GCV treatment in vivo. (**A**) Growth kinetics of NC- and RTM44-tumors in PBS- and GCV-treated mice (100 mg/kg/day) over a period of 14 days. GCV treatment had no impact on outgrowth of NC-tumors, but significantly inhibited growth of RTM44-tumors. Number of tumors per treatment group: NC-PBS, *n* = 5; NC-GCV, *n* = 7; RTM44-PBS, *n* = 4; RTM44-GCV, *n* = 10; statistical analysis: non-parametric *t*-test, adjusted *p*-values corrected for multiple comparisons using the Bonferoni–Dunn method. (**B**) Tumor volume was calculated before (day 0) and at the end (day14) of treatment using the formula V = ½ (L × W^2^). Statistical significance was determined using the Mann–Whitney test, *** *p* < 0.001; ** *p* = 0.01. (**C**,**D**) RTM-tumors exposed to GCV treatment were visibly smaller and weighed significantly less at the end of the treatment compared with tumors of the PBS-treated control group. Mann–Whitney test, ** *p* < 0.01.

To evaluate the feasibility and efficacy of our SMaRT-cancer suicide gene strategy in vivo, RDEB-SCC2 cells stably expressing either RTM44 or the splicing-incompetent NC were injected intradermally into SCID-beige mice. Mice were monitored regularly for tumor development and treated with either GCV (100 mg/kg/day i.p. for 14 days) or PBS when the average tumor volume was ~50 mm^3^. NC-tumors grew at a decreased rate compared with RTM44-tumors (Figure 4A, left and right panel). NC-tumors grew steadily and with similar kinetics in mice that were treated with PBS or GCV (Figure 4A, left panel), indicating that GCV had no effect on NC-tumors. However, the growth of RTM44-tumors was significantly inhibited in mice receiving systemic GCV treatment as compared with those that received PBS (Figure 4A, right). At the end of the treatment, RTM44-tumors excised from mice that had received GCV treatment were visibly smaller (Figure 4B) and weighed significantly less (Figure 4C) than RTM44-tumors that had been exposed to PBS. In contrast, the weight and size of NC-tumors were similar regardless of treatment (Figure 4D). Furthermore, TUNEL staining in whole tumor tissue sections identified areas of increased apoptosis in RTM44- compared with NC-tumors that had been exposed to GCV, indicating that ongoing apoptosis contributed to the tumor growth inhibition observed in these mice (Figure S3). Thus, our results indicate that RDEB-SCC2 cells expressing RTM44 are susceptible to GCV treatment applied systemically, leading to control of tumor outgrowth in a xenograft animal model.

## 3. Discussion

In the present study, we show that an RNA *trans*-splicing molecule (RTM44) is able to reprogram the cancer target gene Ct-SLCO1B3 in order to express thymidine kinase from herpes simplex virus (HSVtk), rendering cancer cells sensitive to treatment with the prodrug ganciclovir in both in vitro and in vivo experiments. This effect is ascribed to a successful *trans*-splicing reaction, followed by expression of an active chimeric HSVtk, and consequent induction of apoptosis, all of which could not be observed in tumor cells containing the *trans*-splicing deficient variant of the RTM (NC). Notably, the efficacy of RTM44 has also previously been demonstrated in a colorectal cancer model [17], thereby highlighting its translatability to other malignancies that express Ct-SLCO1B3. RDEB is a rare genetic skin disease caused by loss of function mutations in the gene *COL7A1*, leading to tissue fragility in skin and mucous membranes. Patients suffer from excessive scarring and chronic open wounds that often become infected and are at high risk of turning malignant [30]. cSCC arises in nearly all RDEB patients and constitutes a life-threatening complication [25]. Notably, these SCCs predominantly develop at sites of chronic wounding, which makes a clear and early diagnosis very challenging. Therefore, new therapeutic approaches that can be applied even at premalignant stages, or in tissue wherein only a few malignant cells are present and could be targeted, is highly warranted in this patient group. SMaRT technology may also be suitable in this context, as it should act only in cells that express the cancer-specific target gene. We designed RTM44 to *trans*-splice to the tumor-specific target pre-mRNA of Ct-SLCO1B3. We have previously shown that Ct-SLCO1B3 is expressed in RDEB-SCC cells [23], (Figure S4A). However, for this technology to be applicable at the pre-malignant stage, its expression in chronically inflamed tissue and wounds in this patient cohort still needs to be investigated.

Furthermore, we were able to detect Ct-SLCO1B3 mRNA levels in late stages of head and neck SCC tissue arising in the normal population (Figure S4B), suggesting that this could be an additional cancer that could be targeted by RTM44. One aspect that needs to be considered is the level of target gene expression in the cancer cells, as this was shown to influence the efficacy of the *trans*-splicing approach. As shown by Sun et al. 2018, colorectal cancer cells with lower levels of Ct-SLCO1B3 RNA expression exhibited reduced susceptibility to this approach compared with cancer cells expressing high levels of the target gene. Therefore, it can be hypothesized that a certain threshold of the target gene needs to be expressed in order to generate sufficient levels of the *trans*-splicing product. Accordingly, the level of Ct-SLCO1B3 expression should be determined for each tumor entity and patient, and these data should be used to select those patients most likely to benefit from this treatment strategy.

In order to advance this strategy further, several aspects of the SMaRT technology still need to be comprehensively investigated. One issue is the potential for off-target events due to unspecific *trans*-splicing. When using RNA *trans*-splicing approaches for gene correction in monogenetic diseases [7,8,31,32,33,34,35,36], it might be possible to overlook off-target events as the introduced gene is not expected to be toxic. However, in the context of suicide gene therapy, any off-target reactions could also induce cell death in normal healthy cells, underscoring the need for comprehensive analysis of such events before moving forward to clinical evaluation. This becomes particularly important when using very potent suicide genes such as diphtheria toxin, where single molecules can kill the target cells [37]. In this case, even a very low frequency of unspecific *trans*-splicing events would cause severe side effects. In contrast to bacterial toxins, whose expression can directly kill cells, the HSVtk/GCV system induces cell death indirectly via DNA replication termination and the activation of DNA damage response [38]. This requires a certain level of GCV-mediated damage signal to induce apoptosis in the cells. Therefore, it has been suggested that low to moderate levels of off-target events may be insufficient to elicit cell death [16]. In line with this, transient transfection of RTM44 into HEK293 cells lacking expression of the target gene Ct-SLCO1B3 did not induce GCV-mediated cell killing (Figure S1), indicating that off-target events are rare or kept under a certain threshold, at least under the conditions investigated.

Additionally, sequence optimization of both vector backbone and RTM is important in order to exclude *cis*-splicing events, or direct expression of the suicide gene from the vector. We have addressed this issue by mutating potential *cis*-splice sites and start codons in the BD that would result in a direct translation from the RTM. Within the HSVtk sequence, several active ATG sites resulted in truncated versions of the suicide gene, which we could confirm to be inactive owing to the lack of the ATP binding site (Figure S2). However, competitive or dominant negative effects of these truncated forms cannot be excluded, especially when the *trans*-splicing reaction is less efficient. Therefore, additional sequence optimization needs to be addressed, and any potential start codon within the HSVtk sequence should be modified in future studies. 

The BD represents another crucial element of the RTM that can be optimised, as it provides specificity by binding to the desired target gene. Off-target events can potentially arise through sequence homology with other, unrelated pre-mRNAs. Notably, for RTM44, no homology between the BD and human genomic sequences other than the target gene SLCO1B3 itself could be detected with NCBI-Blast. However, unspecific *trans*-splicing events cannot be excluded and comprehensive analyses of off-target events induced by the RTM using next-generating sequencing platforms (such as RNAseq) are highly warranted in order to accurately assess both the specificity and efficiency of the approach. 

Finally, the issue of in vivo delivery warrants attention, as this constitutes the bottleneck of any successful gene therapeutic approach. In the present work, we used retroviral vectors to stably introduce the RTM into tumor cells. However, for an in vivo application, the use of retrovirus- or lentivirus-mediated gene delivery systems always harbors the risk of tumor induction in patients owing to possible random integration into the genome [39] or the induction of inflammatory responses [40], and would thus not be the preferable delivery platform for patients. As such, it will be necessary to investigate other gene delivery options with better in vivo applicability. In the context of RDEB-SCC, these tumors are more accessible than other tumor types, which offers the opportunity for local delivery of the RTM. In vivo cell transfection could be enhanced by cloning the RTM cassette into minicircles [41], which, because of their small size and lack of bacterial backbone sequences, show improved transfection efficiencies and in vivo applicability. Furthermore, complexing vectors to highly branched poly (ß-amino ester)s (HPAEs) have already been shown to efficiently deliver genes into skin by injection or even topical application in an RDEB mouse model [42]. Moreover, these can be combined with more mechanical means of gene delivery via microneedle injections [43], gene gun delivery [44], or sonoporation [45]. 

Taken together, SMaRT technology used in a cancer suicide gene therapy approach represents a promising therapeutic strategy. Further studies should focus on optimizing the technology including *trans*-splicing efficiency, specificity, and delivery.

## 4. Materials and Methods

### 4.1. Cell Cultures

RDEB-SCC2 were established from a SCC tumor arising in an RDEB patient (*COL7A1*: c.6269delC/p.P2090fsX116; c.8253_8254delAG/p.R2751SfsX38; [28]). The cells were routinely grown in Green’s medium (DMEM and Ham F-12 (both from Hyclone, Perbio Science, Bezons, France) 2:1) supplemented with 10% FCS II (Biochrom, Berlin, Germany), 2% L-glutamine (Thermo Fisher Scientific, Waltham, MA, USA), and 1% sodium-pyruvate, 0.1416 mM adenine, 5 µg/mL insulin, 0.4 µg/mL hydrocortisone, 47 ng/mL cholera toxin, 0.00137 ng/mL triiodothyronine, and 10 ng/mL EGF (all from Sigma-Aldrich, St. Louis, MO, USA ), at 37 °C and 5% CO_2_. Human embryonic kidney cells (HEK293AD) were purchased from Stratagene (La Jolla, CA, USA) and cultured in DMEM + 10% FC + penicillin/streptomycin.

### 4.2. RTM44

The design and generation of RTM44, NC, and PC was described in detail by Sun et al. [17] and subcloned into the retroviral vector pDON (pDON-AI-2, Takara –Bio, Kusatsu, Shiga, Japan). All resulting vectors (pDON-RTM44 = RTM44, pDON-NC = NC, pDON-PC = PC) were transduced into RDEB-SCC2 cells and further FACS-sorted based on their GFP expression to >90% purity. 

### 4.3. Transient Transfection

HEK293AD cells were cultured in T25 cm^2^ until reaching a confluence of 80% and transfected with Xfect reagent (Clontech, Mountain View, CA, USA), according to the manufacturer’s protocol. Forty-eight hours after transfection, cells were detached and re-seeded in replicate wells of a 96-well plate, and treated with 100 µM GCV the next day. Cell viability was assessed by MTT assay after 72 h of incubation. For transient transfection, trans-splicing constructs encoded in bacterial plasmids (pCDNa3.1) were used.

### 4.4. PCR Analysis

For detection of endogenous *trans*-splicing between SLCO1B3 and the RTM RNA, we performed a one-step RT PCR according to the protocol for Luna^®^ Cell Ready One-Step RT-qPCR Kit Protocol (New England Biolabs, Frankfurt, Germany). Briefly, 100,000 cells were washed with PBS and then lysed with the cell lysis mix, according to the manufacturer’s instructions, and stored at −80 °C. RT-qPCR was performed according to the Luna Universal One-Step RT-qPCR Kit protocol. For each reaction, 4000 cells were used. The following forward primer binding CT-SLCO1B3 exon 1 (fp3680: 5′-TTGGCTTGGGCTCAGAGA-3′) and reverse primer complementary to HSVtk (rp819: 5′-AGATGTTCGCGATTGTCTCGGAA- 3′) were used. PCR detecting GAPDH (fp: 5′- GCC AAC GTG TCA GTG GTG GA-3′; rp: 5′ CAC CAC CCT GTT GCT GTA GCC-3′) served as internal control. PCR conditions: reverse transcription at 55 °C for 10 min followed by an initial denaturation step at 95 °C for 1 min; 40 cycles of denaturation at 95 °C for 10 and extension at 60 °C for 30 s.

Correct integration of the vectors encoding RTM, NC, or PC within the genome of RDEB-SCC was verified by standard PCR analysis using forward primers complementary to the binding domain (BD) (fp3648: 5′-CCTGCTAAAAATCAGCATTCCTAA-3′) or the vector sequence upstream of the BD (fp3681: 5′-GCTTCCTTTGTCCCCAATCT-3′) in combination with the reverse primer binding HSVtk (rp819).

### 4.5. Western Blot Analysis

Whole cell lysates were prepared from each RDEB-SCC cell line using RIPA buffer (Santa Cruz Biotechnology, Dallas, TX, USA) supplemented with 0.1% protease inhibitor cocktail (Roche, Basel, Switzerland). Samples were mixed with 4× NuPUGE LDS sample buffer (Invitrogen, Carlsbad, CA, USA) with 3% β-Mercaptoethanol and separated on a NuPAGE 4–12% Bis-Tris gel (Invitrogen) under denaturing conditions. Afterwards, the proteins were electroblotted onto a PVDF membrane (GE Healthcare, Buckinghamshire, UK) and blocked with 5% skim milk in TBS/0.1% Tween 20 for 1 h at RT. The membrane was incubated with monoclonal mouse-anti-Flag-tag antibody (Wako 1:1000) in blocking buffer over night at 4 °C followed by incubation with the secondary HRP-labeled anti-mouse IgG antibody (DAKO 1:200) for 1 h at RT. Alpha actinin staining served as loading control using a polyclonal rabbit-anti-alpha-actinin (Santa Cruz Biotechnology, 1:1000) antibody followed by incubation with HRP-labeled anti-rabbit IgG antibody (DAKO, Vienna, Austria; 1:200). Protein bands were visualized using Immobilon Western Chemiluminescence HRP Substrate (Merck Millipore, Darmstadt, Germany) and ChemiDoc XRS system (Bio-Rad, Munich, Germany). 

### 4.6. TUNEL Staining

Terminal deoxynucleotidyl transferase dUTP nick end labeling (TUNEL) assay was performed to highlight apoptotic cells in tumor sections using the In Situ Cell Death Detection Kit TMR red (Roche, Basel, Switzerland), according to the manufacture’s protocol. Briefly, tissue sections were fixed with 4% paraformaldehyde for 20 min at RT and permeabilized with PBS containing 0.1% Triton X-100 and 0.1% BSA. Afterwards, tissue sections were stained with 50 µL TUNEL reaction mixture for 1 h at RT. Cell nuclei were staining using 4’, 6-diamidino-2-phenylindole (DAPI). Immunofluorescence on whole tissue sections was assessed with the Olympus VS120-LD100 slide loader system (Olympus, Tokyo, Japan).

### 4.7. MTT Assay and Confluency Measurement

Cells were seeded into a 96-well plate at a density of 5000 cells/well in 100 µL medium and incubated at 37 °C for 24 h. The next day, 100 µL medium supplemented with ganciclovir (10–200 µM) was added. Cells grown in medium only served as negative control. After 72 h of incubation, 25 µL of MTT stock solution (5 g/L Thiazolyl Blue Tetrazolium Bromide in PBS, Abcam, Cambridge, UK) was added and the plate was incubated for 1 h at 37 °C. Afterwards, the supernatant was aspirated and cells were lysed by adding 100 µL DMSO/glycine solution (6 Vol DMSO + 1 Vol 0.1 M glycine/NaOH pH 10.2). The plate was incubated for 10 min at RT on a plate shaker at 500 rpm. Finally, absorbance (reduction of MTT to formazan) was measured at 492 nm/670 nm using the TECAN Spark M10 plate reader (TECAN, Grödig, Austria). For confluence measurement, cells were seeded into a 96-well plate at a density of 2000 cells/well and treated as described above. After 72 h of treatment, cell confluence within the wells was measured and brightfield pictures were acquired using TECAN Cyto M10 plate reader (TECAN, Grödig, Austria).

### 4.8. Animal Model

Immunodeficient SCID beige mice (CB17.Cg-*Prkdc^scid^Lyst^bg-J^*/Crl) were purchased from Charles River (Sulzfeld, Germany) and maintained in the SPF animal facility of the Paracelsus Medical University Salzburg, Austria. All animal experiments were conducted in accordance with the guidelines of the facility and with approval of the local regulatory committee (license number: TVA 20901-TVG/72/7-2014).

RDEB-SCC2 (3.5 × 10^6^ cells) carrying either RTM44 or NC were injected intradermally into the shaved abdomen of the mice. After the tumors reached a volume of 50 mm^3^, ganciclovir (100 mg/kg) was administered intra peritoneally (i.p.) every day. Tumor volume was measured every second day with a digital caliper and tumor volume was calculated using the formula V = ½ × length(L) × width(W)^2^. After two weeks of treatment, mice were sacrificed and tumors were excised for further analysis.

### 4.9. Statistical Analysis

GraphPad Prism software v. 9 (GraphPad, San Diego, CA, USA) was used for statistical analysis. The data were analyzed for normal distribution using the Kolmogorov–Smirnov test, D’Agostino and Pearson omnibus normality test, and Shapiro–Wilk normality test. Unpaired t-test (two-tailed) was used when data in two groups were normally distributed. When the data to be compared were not normally distributed, the Mann–Whitney test (two-tailed) was performed. The results were considered significant at * *p* ≤ 0.05, ** *p* ≤ 0.01, and *** *p* ≤ 0.001.

## 5. Patents

J.W.B., U.K., and C.G.-G. are inventors of patent PCT/EP2013/072823, US9655979B2, and EP2914721, RNA *trans*-splicing molecule (RTM) for the use in cancer treatment. T.F. is listed as an inventor of Ct-SLCO1B3 patents JP5901046, US9115405, and US2012014977. 

## Data Availability

Data sharing not applicable as no datasets have been generated or analyzed in this study.

## Supplementary Materials

The following are available online at https://www.mdpi.com/article/10.3390/ijms23010575/s1.
