# Supplementary material for: Evaluating a Targeted Cancer Therapy Approach Mediated by RNA trans-Splicing In Vitro and in a Xenograft Model for Epidermolysis Bullosa-Associated Skin Cancer
Source: Int J Mol Sci. 2022 Jan 5;23(1):575. doi: 10.3390/ijms23010575 (PMC8745581; doi:10.3390/ijms23010575)
Supplement: Supplementary file 1 [file ijms-23-00575-s001.zip › ijms-1461829-supplementary.pdf]

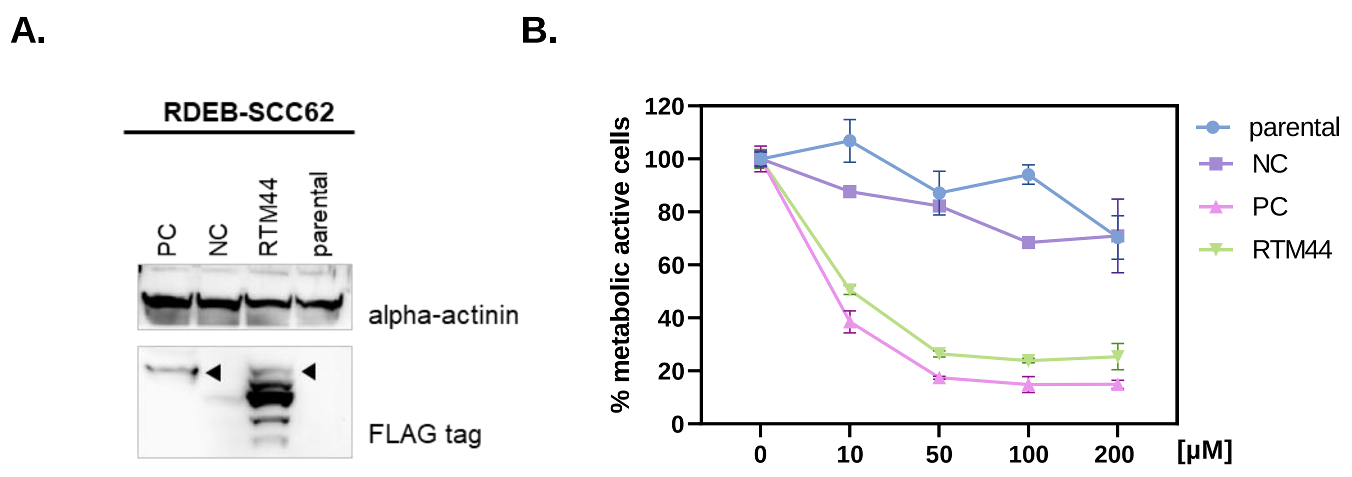

Figure S1: *Trans*-splicing in RDEB-SCC62 cells. (A) Detection of *trans*-splicing product between Ct-SLCO1B3 and HSVtk in RDEB-SCC62 cells by western blot analysis. The triangles indicate the fusion protein (42 kDa). (B) MTT assay demonstrating sensitivity of PC and RTM44 transduced RDEB-SCC62 cells to increasing concentrations of ganciclovir (0 – 200 µM).

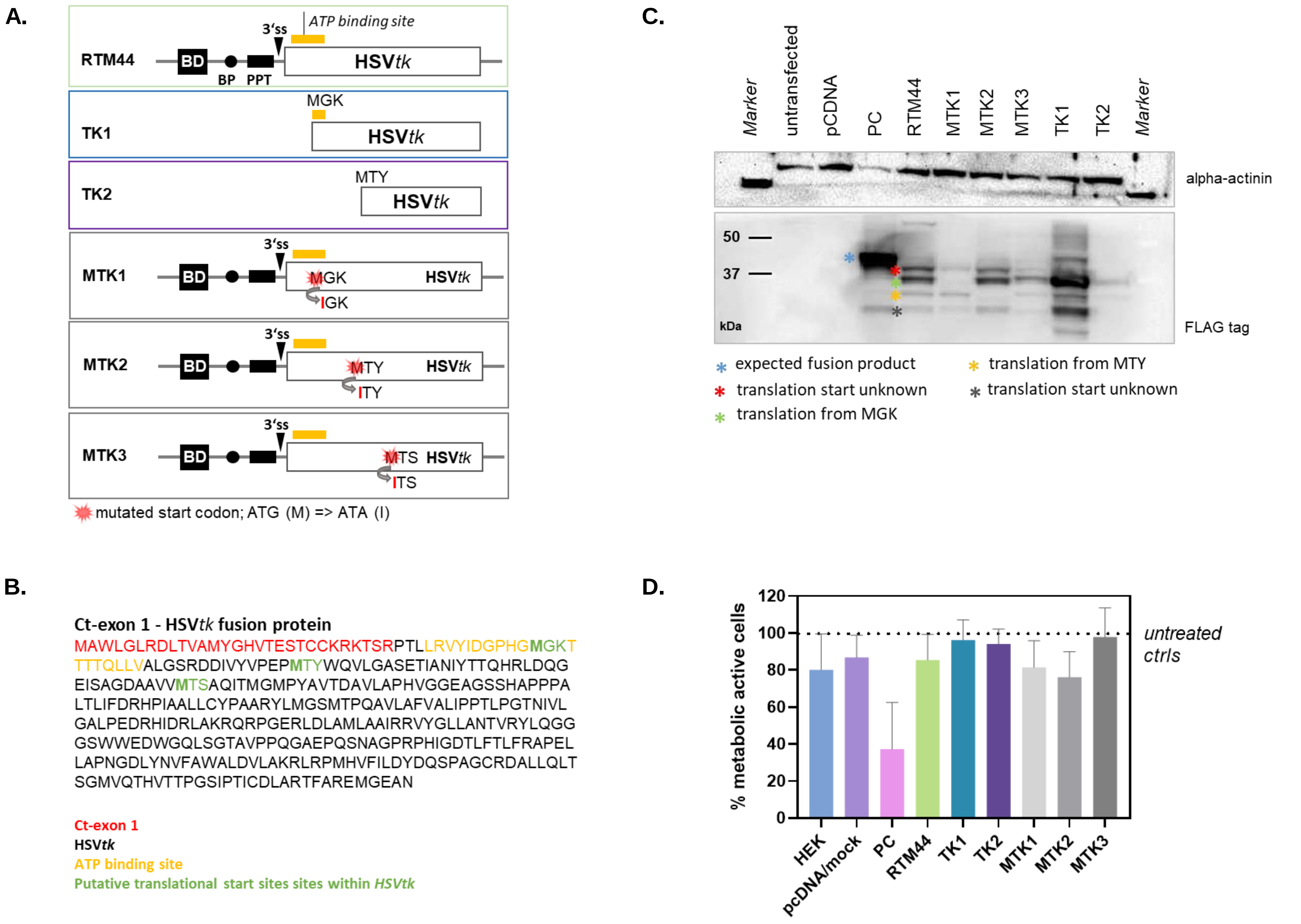

Figure S2: Evaluation of different variants of HSVtk

(A) Schematic presentation of RTM44, TK1 and TK2. RTM44 consists of a binding domain (BD), splicing elements (BP, PPT, 3' ss) and the coding sequence of HSVtk. TK1 starts with the first ATG within HSVtk (MGK; MW 37.3 kDa), TK2 starts with the second ATG (MTY; MW 34.5 kDa). Furthermore, we mutated each of three potential start codons in the RTM44 vector separately (MTK1, MTK2 and MTK3). (B) Amino acid sequence of the predicted chimeric protein resulting from accurate *trans*-splicing. ATP binding site of HSVtk is highlighted in yellow, putative start codons are marked in green. (C) Western blot analysis of truncated and mutated versions of HSVtk. Three different ATGs (MGK, MTY and MTS) are located in the HSVtk and are in frame with the fusion-transript start codon. Blue indicates the expected fusion protein (42 kDa), green/yellow asterisks mark proteins translated from MGK and MTY respectively and red/grey asterisks highlight proteins of unknown origin. (D) MTT assay of HEK293 cells transiently transfected with plasmid encoding the different variants of HSVtk.Cells were treated with 100 µM GCV for 72 hours. Only PC transfected cells were sensitive to GCV treatment. Each experiment was carried out in quadruplicates. Mean ± SD of 2 experiments is shown. Thus, proteins resulting from downstream translational start sites are suggested not to be active since they would lack the ATP binding site.

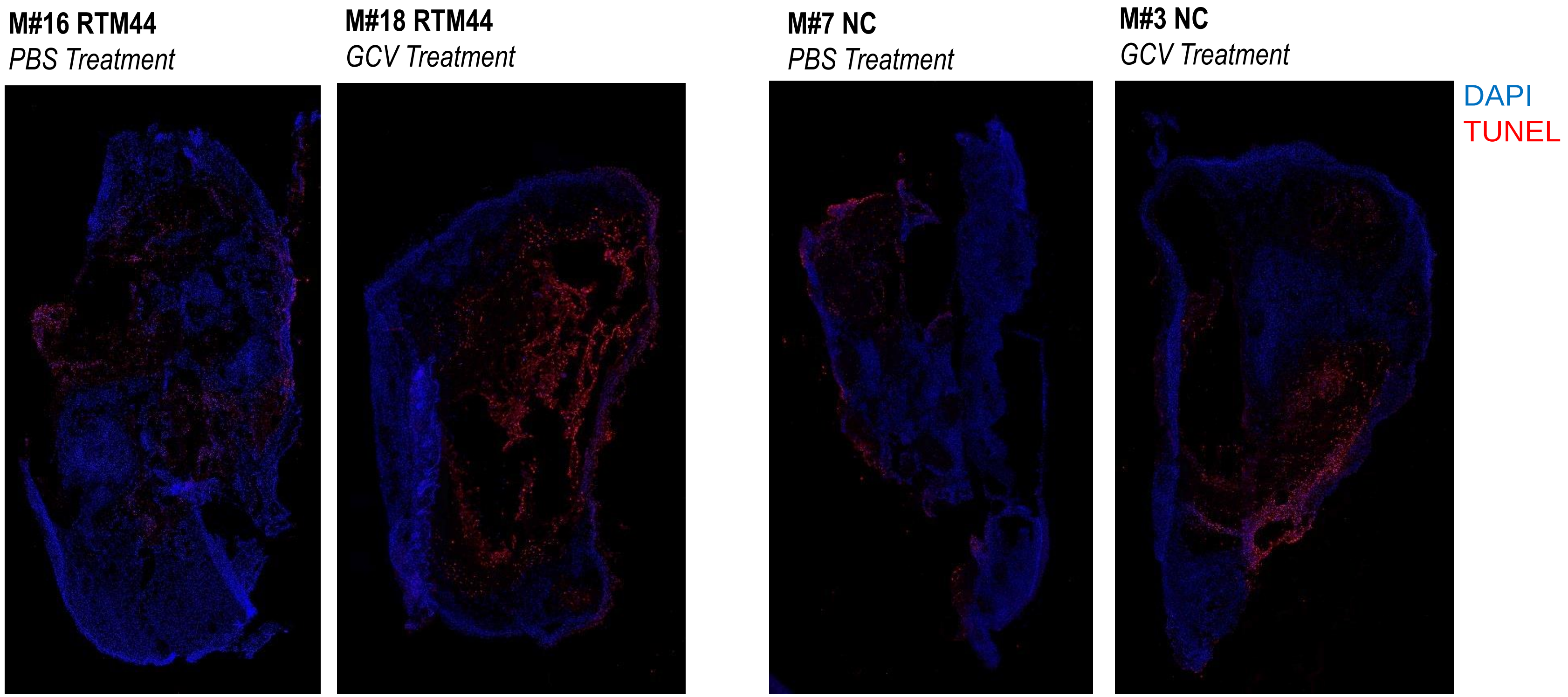

Figure S3: Detection of apoptosis in tumor sections

Terminal deoxynucleotidyl transferase dUTP nick end labeling (TUNEL) assay was performed to highlight apoptotic cells in tumor tissue from SCC/RTM44 (mouse #16, #18) and SCC/NC (mouse #7, #3) bearing mice using the In Situ Cell Death Detection Kit TMR red (Roche) according to the manufacturer's protocol. Mice were either treated with GCV or PBS for 14 days and tumors excised at the end of treatment for analysis. Cell nuclei were stained with 4', 6-diamidino-2-phenylindole DAPI. Immunofluorescence of whole tissue sections were assessed with Olympus VS120-LD100 slide loader system.

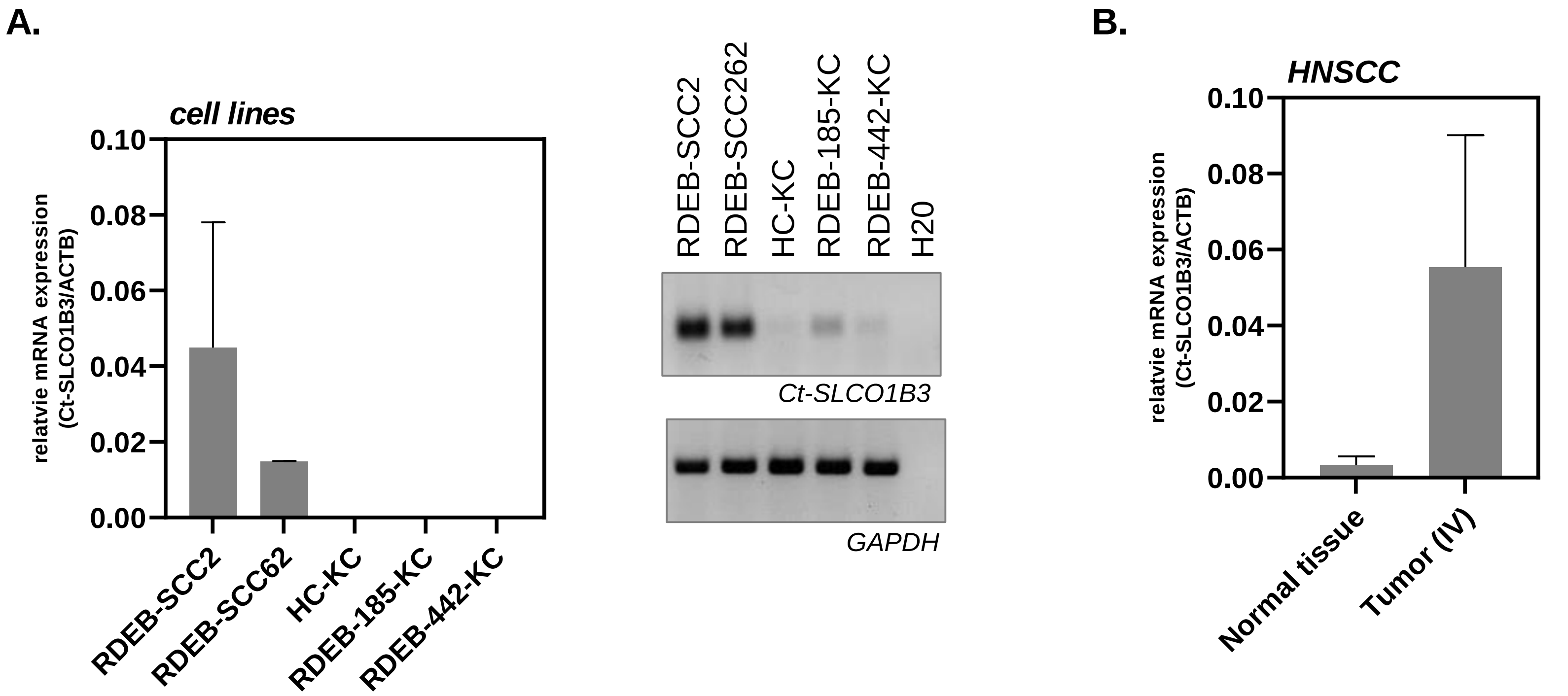

Figure S4: Expression profile of Ct-SLCO1B3. (A) Relative expression ($2^{-\text{delta ct values}}$) of Ct-SLCO1B3 in RDEB–SCC lines, normal human keratinocytes (HD-KC) and RDEB keratinocytes (RDEB-185-KC[1]) by sqRT-PCR analysis (left). Agarose gel electrophoresis of sqRT-PCR products (right). (B) Increased levels of Ct-SLCO1B3 were also detected in total RNA isolated from several head and neck SCC tumor biopsies (stage IV, n = 6) compared to normal tissue (n = 8) by sqRT-PCR (TissueScan, Head and Neck Cancer cDNA Array, Origene).

[1]Reference: Kocher et al, *JID* 2020.
